# An Efficient Neural Network Design Incorporating Autoencoders for the Classification of Bat Echolocation Sounds

**DOI:** 10.3390/ani13162560

**Published:** 2023-08-08

**Authors:** Sercan Alipek, Moritz Maelzer, Yannick Paumen, Horst Schauer-Weisshahn, Jochen Moll

**Affiliations:** 1 Department of Physics, Goethe University of Frankfurt, 60438 Frankfurt am Main, Germany; maelzer@physik.uni-frankfurt.de (M.M.); moll@physik.uni-frankfurt.de (J.M.); 2 Frankfurt Institute for Advanced Studies, 60438 Frankfurt am Main, Germany; paumen@fias.uni-frankfurt.de; 3 Freiburg Institute of Applied Animal Ecology, 79110 Freiburg, Germany; schauer-weisshahn@frinat.de

**Keywords:** bat echolocation sound analysis, bat species classification, machine learning, convolutional neural network, autoencoder, clustering, animal population monitoring

## Abstract

**Simple Summary:**

Bats play a crucial role as bioindicators of environmental changes, making their monitoring highly valuable. In particular, wind energy plants have been found to cause significant fatality rates among bats, as well as birds, mainly through direct collision with the rotor blades or through barotrauma effects. However, the manual identification and classification of bats through their echolocation sounds is an expensive and time-consuming process. To address this issue, we present an automated analysis pipeline applied to a large dataset recorded over a period of two years in a wind test field. This work proposes various statistical methods based on convolutional neural networks and clustering techniques to examine the relationship between background noise and bat echolocation sounds. In addition, the methodology performs classification at both the genus and species levels, with a high accuracy for most bat classes.

**Abstract:**

Bats are widely distributed around the world, have adapted to many different environments and are highly sensitive to changes in their habitat, which makes them essential bioindicators of environmental changes. Passive acoustic monitoring over long durations, like months or years, accumulates large amounts of data, turning the manual identification process into a time-consuming task for human experts. Automated acoustic monitoring of bat activity is therefore an effective and necessary approach for bat conservation, especially in wind energy applications, where flying animals like bats and birds have high fatality rates. In this work, we provide a neural-network-based approach for bat echolocation pulse detection with subsequent genus classification and species classification under real-world conditions, including various types of noise. Our supervised model is supported by an unsupervised learning pipeline that uses autoencoders to compress linear spectrograms into latent feature vectors that are fed into a UMAP clustering algorithm. This pipeline offers additional insights into the data properties, aiding in model interpretation. We compare data collected from two locations over two consecutive years sampled at four heights (10 m, 35 m, 65 m and 95 m). With sufficient data for each labeled bat class, our model is able to comprehend the full echolocation soundscape of a species or genus while still being computationally efficient and simple by design. Measured classification F1 scores in a previously unknown test set range from 92.3% to 99.7% for species and from 94.6% to 99.4% for genera.

## 1. Introduction

The current geological epoch in which humankind is rapidly changing the global landscape is coined the Anthropocene. With the increasing demand for resources and living space, humans have a negative impact on Earth’s climate and biosphere [1,2,3]. Wind energy plays an essential role in improving climate health. On the other hand, wind turbines cause measurable harm to ecosystems of flying animals like birds and bats. Bats roosting and foraging close to wind energy plants are likely to collide with rotor blades or experience barotrauma, leading to high fatality rates [4,5,6,7,8,9,10,11,12]. In fact, many bats are highly endangered species and susceptible to anthropogenic changes of their ecosystems [13,14]. Recent evaluations have revealed that many species provide significant monetary benefits to the agricultural industry [14]. Moreover, because they inhabit a multitude of different areas around the world and given their innate sensitivity to environmental changes, bats are considered essential bioindicators [15,16].

Acoustic surveillance of bat activity is of great importance for mitigation and curtailment systems of wind turbines [17,18] and for general population monitoring apart from wind energy applications [19]. Non-invasive acoustic monitoring of bat activity has a long history of improvements regarding hardware, data management and analysis methodology [19]. Continuous acoustic monitoring of bat activity generates large volumes of data. The manual labeling process of such data, mainly done by human experts, is a time-consuming and costly endeavor. To enhance the quality and consistency of bat conservation, there is a need for fast and robust automated detection systems similar to Sonobat [20] and Kaleidoscope [21].

Traditional sound detection methods apply different thresholds for frequency and amplitude or quantify the areas of smooth frequency changes. Many such methods struggle to reliably differentiate between all species or genera [22]. Conventional species identification, on the other hand, relies upon hand-tailored features, like various frequency levels, call durations and interpulse intervals [22]. Common statistical models for species classification include random forests [23,24,25,26,27], k-nearest neighbor [27], support-vector machines [23,27,28] and discriminant function analysis [23,27,29].

After the first significant triumph of convolutional neural networks (CNNs) as image classifiers in 2012 [30], such deep learning models became the state of the art in a multitude of disciplines and domains. A CNN can be used to detect bat sounds within audio recordings or even identify their species by learning spectrograms computed via a short-time Fourier transform (STFT) from such audio data. The great advantage of (deep) neural networks is their strong ability to find meaningful patterns inside the training data, making the need for feature engineering mostly irrelevant. The first deep-learning-based model to detect bat echolocation sounds from audio data was Bat Detective [31], which was recently expanded into a joint model that performs bat sound detection and species identification [32]. The first iteration consists of two versions of a CNN trained on single bat pulses. For the prediction step, it moves in a sliding-window fashion along the spectrogram in order to detect individual pulses with a window size of 23 ms [31]. The second iteration is a CNN performing complete end-to-end bounding-box detection for spectrograms of less than two seconds [32]. This approach allows a model to learn temporal dependencies between consecutive bat pulses. Additional works on bat species identification with CNNs can be found in [33,34,35,36,37]. The works of Chen et al. [33], Kobayashi et al. [34] and Schwab et al. [35] are involved the use of small windows of 20 ms, 27 ms or 10 ms in size to detect individual bat pulses, similar to the work of Aodha et al. [31]. In contrast, Tabak et al. [36] trained their CNN with noiseless plots of five to fifteen consecutive pulses. Finally, Zualkernan et al. [37] used STFT-based images of 3 s audio segments to train their CNN model.

In this work, we propose a CNN-based approach that expands the developments of Paumen et al. [38] regarding the behavioral analysis of used models and datasets. We also use the same CNN structure to classify Mel-frequency cepstral coefficients (MFCC) of 1 s audio segments containing noise, either with or without bat sounds. With the additional help of an unsupervised geometric analysis via a convolutional autoencoder followed by UMAP clustering, we provide useful insights for model and dataset choice before training the CNN classifier. This auxiliary process is similar to that proposed by Kohlsdorf et al. [39], who trained a convolutional recurrent autoencoder followed by k-means clustering and T-SNE 2D projection. A related approach to gain additional knowledge about the data by k-means clustering was proposed by Yoh et al. [24]. Our approach involves the use of three separately trained classifiers in order to perform bat sound detection, genus identification and species identification. Combined with human expert domain knowledge, this also allows for more authentic interpretation of the classifiers behavior. Compared to Paumen et al. [38], our data is extended by three additional collections of data, which allows for the comparative analysis of two collections with different locations in the same year and two collections from different years but at the same location. All four collections were gathered from the NatForWINSENT wind test field in Germany between 2019 and 2020. Most of the previously named works using CNNs and other machine learning models examined datasets with only individual bat species, whereas our four data collections contain individual species, genera, groups of similar echolocation behavior and various types of stationary and non-stationary noise recordings. This offers new insights in the handling of sophisticated bioacoustic data. We claim our data processing pipeline to be novel in the context of bioacoustic data, since we could not find any previous study that combines unsupervised learning with supervised learning techniques in a seamless pipeline. Additionally, no previous study on bat echolocation sound analysis has elaborated on the presence of background noise recordings in such detail as this work. Furthermore, with the full code being open-source, an educational template is provided to the community of biologists to foster collaboration. In the field of automated bat sound analysis, open-source code is rare in comparison to commercial tools like Sonobat [20], Kaleidoscope [21], BatCallID [40], BatSound [41] and Anabat Insight [42].

## 2. Materials and Methods

The following section describes the acquisition process of the acoustic data, its preprocessing and the methods used for a machine-learning-based analysis. The complete code is written in Python 3.7 with the Pytorch 1.9.0 deep learning framework with CUDA 11.3 and can be downloaded from Mendeley Data under http://doi.org/10.17632/9x2g6dsbtv.1.

### 2.1. Data Acquisition

#### 2.1.1. Location and Measurement Devices

The acoustic monitoring of bat activity was deployed according to the German Federal Agency for Nature Conservation (German: Bundesamt für Naturschutz, BfN) under the funded R+D project “Implementing nature conservation research at the land-based wind test site” (FKZ 3518 86 0100). Data acquisition happened on a wind test site that is located in the Swabian Alb near Geislingen an der Steige, east of Donzdorf. Two measuring masts with a height of 100 m were equipped with four microphones each installed at heights of 10 m, 35 m, 65 m and 95 m. Both masts were located within about 350 m proximity of each other while; one was placed in the northwest of the testing area, and the other was placed in the northeast of the testing area. The recording devices, including the microphones, were directed to the west on both masts. For all eight installed recording devices, the BATmode S+ system from bat bioacoustictechnology GmbH was used. The BATmode S+ system covers a bandwidth of 300 kHz with 16bit resolution and stores recordings digitally on a hard disk. Thus, the data acquisition process was fully non-invasive, i.e., audio data were gathered without impacting the bats’ ecosystem after the initial system installation.

#### 2.1.2. Labeling by Human Expert

Recorded data from the BATmode S+ system were evaluated and labeled by a human expert (Horst Schauer-Weisshahn) using BATscreen PRO software from bat bioacoustictechnology GmbH. The software provides several options to review audio data in form of a spectrogram and help the human expert to classify the recordings with respect to frequency thresholds, power density, interpulse intervals and call duration. It is important to mention that the labeling process by the human expert was purely based on these acoustic data. Due to interspecific and intraspecific variations for different genera and species, a flawless acoustic distinction is not possible without the help of other acquisition methods [43,44]. Hence, a certain configuration of species, genera and groups was defined in the labeling process, as shown in Table 1. Cases of multiple species within the same audio recording were too sparse for proper learning and were discarded before model optimization.

### 2.2. Data Preprocessing

Both the unsupervised and supervised learning tasks were performed with a dataset split ratio of 60:20:20 for training, validation and test data, respectively. The raw audio recordings, each stored in a wav file, were processed with the librosa python package. STFT (short-time Fourier transform) was conducted with 2048 samples per FFT window (fast Fourier transform) for a sampling rate of 300 kHz. This led to a time resolution of 6.8 ms per window. The overlap of two consecutive windows was 75%.

#### 2.2.1. Distributions of Bat and Noise Data

The acoustic data used in this work were gathered through the years of 2019 and 2020 for both measuring masts as explained in Section 2.1.1. With one mast measuring the northeast area and one covering the northwest area, four different data collections are defined by location and year, i.e., east 2019, west 2019, east 2020 and west 2020. Our data are highly imbalanced within a collection, i.e., among species and genera, but shows general consistency in terms of imbalance between years for the same location (Table 2).

Two aspects were be considered. First, in contrast to Paumen et al. [38], we used the full length of each audio recording containing bat sounds, ranging from 2 to 16 s. Taking into account the post-trigger time of the microphone, each audio recording of a bat was recycled for the first 80% of its full length to guarantee at least one pulse per 1 s segment. A 1 s segment usually contains more than one pulse based on the interpulse interval of the recorded individual. As a consequence, the number of 1 s audio segments is not consistent with the number of bats being tracked by the BATmode S+ system. Still, recycling the full audio recording is an intuitive way to increase the natural sample size and, therefore, the quality of the data. Such a method does not replace the relevant pulse variation among individuals of a species, genus or group needed for a high-quality dataset but aids in learning pulse variations within single individuals, since the echolocation behavior in a single sequence alters over time. Second, we did not split the distribution of bat recordings into the four measurement heights because, in contrast to Paumen et al. [38], our incentive was to investigate the impact of differently positioned measurement devices and distant acquisition time frames. Results reported by Paumen et al. [38] also indicate that a model trained at a single measurement height is not robust in species and genus identification of data recorded at other heights at the same location and in the same year. One explanation is that different species forage and socialize at various heights, causing different sound profiles based on the distance to each microphone due to its trigger thresholds. Therefore, it seems reasonable to handle each location–year pair as a complete unit by combining all heights into one dataset.

The noise distribution is purposely listed for each height to show the inconsistent amount of gathered noise information per height in each collection (Table 3). Visualizations of various noise patterns are shown and evaluated in Section 2.2.3.

#### 2.2.2. Spectrograms of Bat Recordings Superimposed by Noise

**Figure 1:**
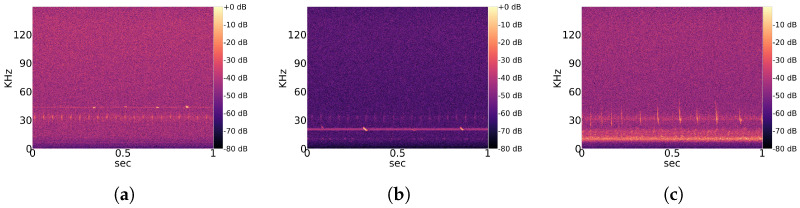
Selection of ordinary echolocation sounds from different genera and common stationary noise. The potential of overlapping pulse shapes and noise patterns is demonstrated. (**a**) Echolocation sound of genus Pipistrellus; (**b**) Echolocation sound of genus Nyctalus; (**c**) Echolocation sound of genus Myotis.

Typical echolocation sounds of the genera Pipistrellus, Nyctalus and Myotis are shown in Figure 1. The signal around 30 kHz in Figure 1a,b is some kind of stationary noise that occurs in many recordings. In Figure 1c the most prominent noise signal is between 10 kHz and 20 kHz, and the pulses from the Myotis bat overlap with the weaker noise signal around 30 kHz.

#### 2.2.3. Spectrograms of Noise Recordings without Bat Sounds

**Figure 2:**
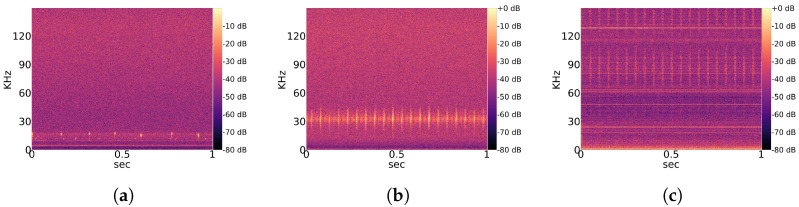
Overview of characteristic *stationary* noise patterns across the total dataset. (**a**) Characteristic noise from East 2019; (**b**) Characteristic noise from West 2019; (**c**) Characteristic noise from West 2020.

**Figure 3:**
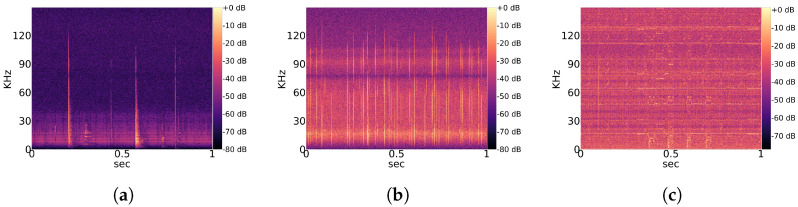
Overview of characteristic *non-stationary* noise patterns across the total dataset. (**a**) Characteristic noise from East 2019; (**b**) Characteristic noise from East 2020; (**c**) Characteristic noise from West 2020.

Observed noise recordings are divided into stationary and non-stationary types. Stationary indicates the regular occurrence of signal patterns with a similar structure over time. A partial overview of regularly measured characteristic stationary noise patterns is provided in Figure 2, and that of non-stationary patterns is shown in Figure 3. Those noise patterns are apparently not linked to the location, year and height of measurement. They do not appear to be of natural or biological cause. Since a majority of bats experience their environment through auditory sensation, they rely on echolocation sounds with regular pulses (Figure 1). Therefore, it is likely that mathematical detection models confuse noise patterns, such as in Figure 2a with Pipistrellus pulses or the patterns shown Figure 2b with Myotis pulses. This means that the present work considers real-world conditions represented by noise-related signal complexity.

### 2.3. Theory

#### 2.3.1. Analysis Pipeline

**Figure 4:**
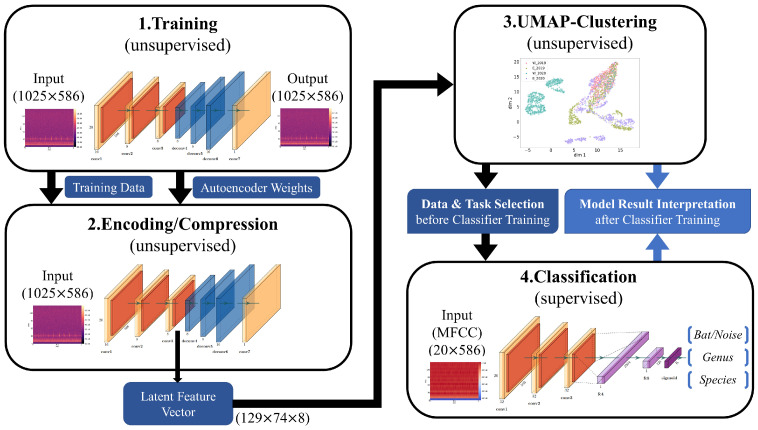
Analysis pipeline that combines unsupervised learning with supervised learning. A convolutional autoencoder is used to learn an efficient representation of the dataset of linear spectrograms. This representation, called a latent feature vector, is then fetched from the encoder part after training the autoencoder. Clustering is performed on these vectors for the dataset, which aid in convergence and smooth the cluster shapes, since the autoencoder can remove irrelevant data. Insights from the geometrical analysis via clustering help in the setup of classification tasks and their interpretation after training.

**Figure 5:**
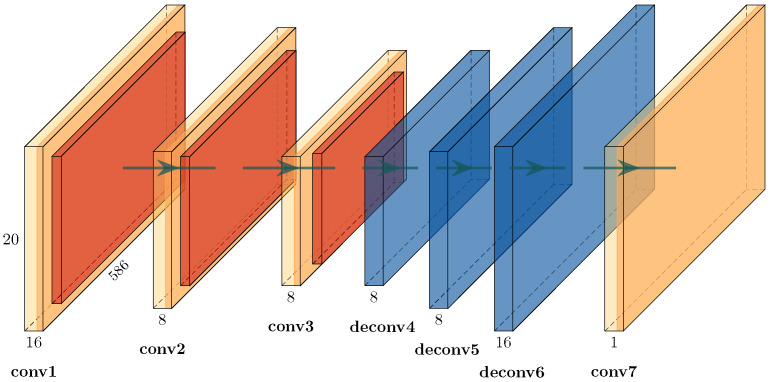
Detailed view of the convolutional autoencoder depicted in Figure 4. The encoder part of three consecutive convolutional layers is used as a feature extractor. The decoder part of three consecutive deconvolutional layers is used for image reconstruction. All pairs of layers have an intermediate rectified linear unit (ReLU), a max-pooling layer and a batch-normalization layer. Final convolution is used to retrieve a single-channel representation of the input. The number of channels is shown above the name of each layer. The size of the convolution filter matrix is 3 × 3 for each layer, except in *conv7* (4 × 4). Convolutional layers have a stride of 1, and deconvolutional layers have a stride of 2. Pooling is 2 × 2 with a stride of 1.

Our complete analysis pipeline is illustrated in Figure 4. First, a convolutional autoencoder is trained on linear spectrograms with a resolution of 1025 × 586 with frequency by time in an unsupervised manner. Each iteration of the training phase it is optimized to reconstruct the current input image. If an autoencoder is optimized properly, gains a considerable understanding of the data and learns to separate meaningful patterns from noise. This process is enforced by the encoder–decoder structure of the network. The encoding process reduces the amount of information passed from layer to layer until the bottleneck. From there on, the decoder part learns to recreate the information from the bottleneck until it reaches the input data resolution shown in Figure 5. As a consequence, the latent feature vector from the bottleneck is an efficient representation of the current input image with dimensions of 129 × 74 × 8.

The reconstruction quality of the autoencoder is evaluated with two common full-reference metrics (Section 3.1.2). One is the peak signal-to-noise ratio (PSNR), which compares the pixel values of the input image to those of the reconstructed image. It ranges from 0 to infinity on a logarithmic scale, where 30 dB or higher is considered good for 8-bit images [46]. The other metric is the structural similarity index measure (SSIM), which is designed to evaluate image quality similar to human perception by focusing on features like luminance, contrast and structure [47]. It ranges from 0 to 1, with 1 being the ideal.

Second, after the autoencoder converges well on the data, the same data are again passed through the encoder of the network. The emitted latent feature vectors are then fed into a UMAP projection to cluster the data. The motivation for using latent feature vectors is their reduced data size compared to the raw spectrograms (reduction of ∼87%), leading to much faster convergence of the clustering algorithm without a loss of the core information. Since the geometry of the data decides which UMAP hyperparameters fit the data the best, a thorough grid search needs to be performed for each investigated composition of datasets. With the requirement of running a grid search for UMAP hyperparameters, this reduction becomes quite advantageous.

**Figure 6:**
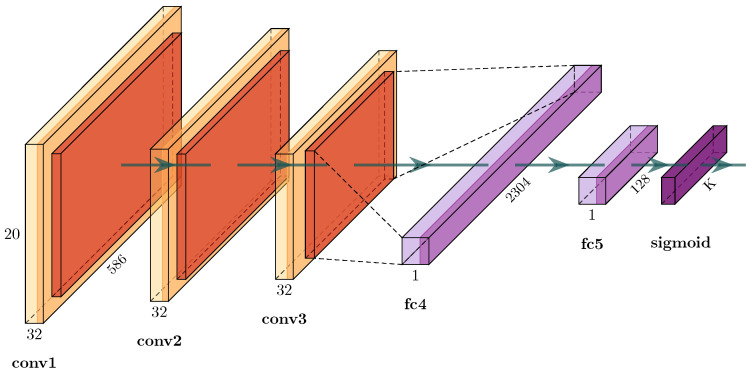
Detailed view of the convolutional network for image classification shown in Figure 4. The feature extractor of three consecutive convolutional layers with intermediate rectified linear units (ReLUs), a max-pooling layer and a batch-normalization layer. Classifier with two consecutive fully connected layers and sigmoid as nonlinear activation function. The number of channels is shown above the name of each layer. The size of the convolution filter matrix is 3 × 3 for *conv1* and *conv2* and 2 × 2 for *conv3*, all with a stride of 1; pooling is 2 × 2 with a stride of 2. *K* is the number of output classes.

Insights gained from such clustering help to decide which properties of the dataset should be investigated and how different sources of data should be composed (i.e., height, year and location) for each classification task (bat/noise, genus, species, etc.). Moreover, geometrical relationships between clustered datasets are harnessed to interpret the behavior of our supervised convolutional neural network models (Figure 6) and to better expose potential limitations of the data.

As for the classification part of the analysis pipeline, the CNN classifier of each task is fed with MFCCs instead of linear spectrograms, which reduces the data size by ∼98%. This allows for more efficient model training without a significant loss in predictive ability. The resolutions of both input formats are shown in Figure 4.

The F1 score is our main score metric used to evaluate the performance of the classification model presented in Section 3.2 and Section 3.3 and is defined in Equation (1). The abbreviations are defined as follows: TP, true positive; FP, false positive; FN, false negative.

(1) $$F_{1}=\frac{2\times \mathrm{precision}\times \mathrm{recall}}{\mathrm{precision}+\mathrm{recall}}=\frac{2\times \mathrm{TP}}{2\times \mathrm{TP}+\mathrm{FP}+\mathrm{FN}}$$

The next section is divided into three subsections following the analysis pipeline presented in Figure 4. First, an unsupervised learning approach is applied on the data presented in Section 3.1, focusing on pure noise recordings and their potential similarities with bat pulses on the genus level. Second, the impact of measurement years and locations on supervised models for bat/noise, genus and species classification are investigated in Section 3.2. Finally in Section 3.3, a single classification model combining all data collections is trained for genus and species identification in order to verify the model’s learning capabilities.

## 3. Results

To train the convolutional autoencoder, we used the ADAM optimizer with a learning rate of 0.0001 and a batch size of 1. For the three supervised learning tasks, we also trained the CNN using ADAM with a learning rate of 0.0001 but with a batch size of 32 in this case. The autoencoder converged sufficiently within 10 epochs for the given optimizer settings. Our CNNs for bat/noise classification, bat genus classification and bat species classification required 10 epochs, 50 epochs and 25 epochs, respectively.

### 3.1. Unsupervised Learning

#### 3.1.1. Clustering of Noise Recordings

**Figure 7:**
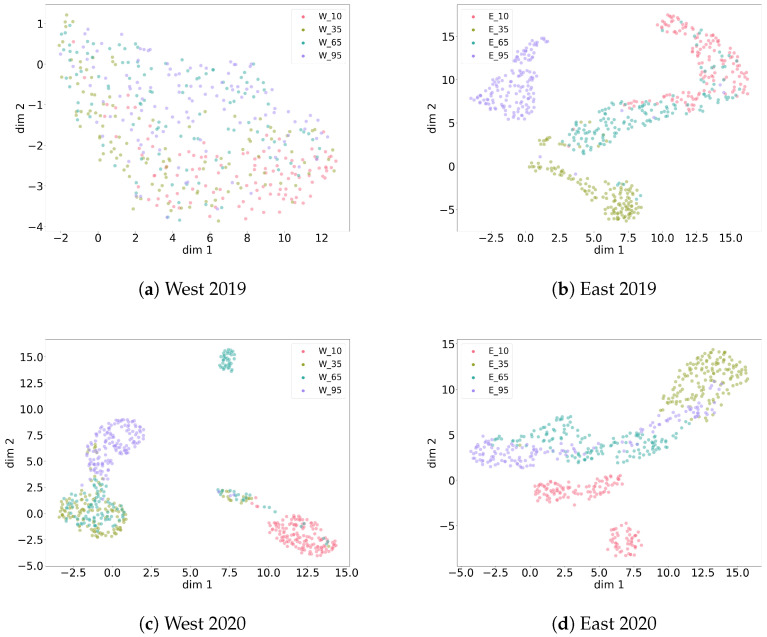
Overview of several UMAP clusterings of noise recordings from four data collections, each over four measurement heights. Different heights correspond with different noise types. West 2019 shows the highest similarity between its measurement heights.

**Figure 8:**
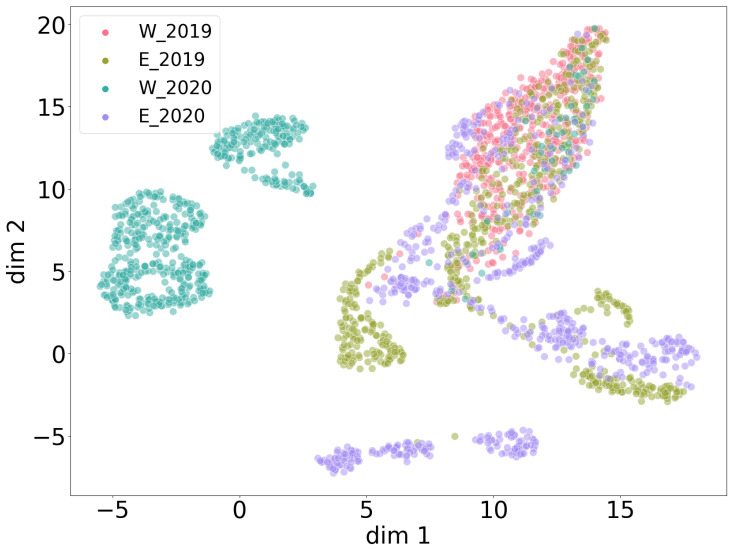
Global UMAP clustering with noise recordings from all four data collections. West 2019, east 2019 and east 2020 show stronger global similarity, while west 2020 and parts of east 2020 show significant differences relative to the other clusters.

**Figure 9:**
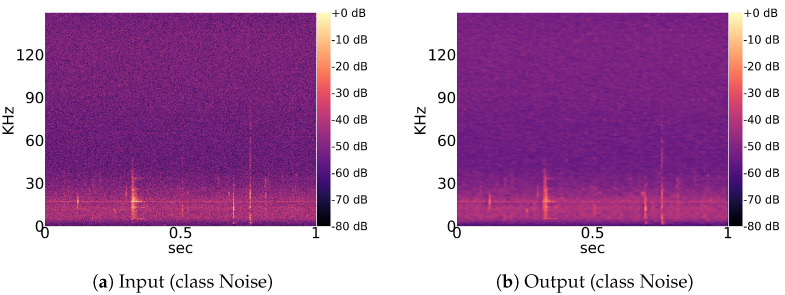
Input and output example for a pretrained convolutional autoencoder. The autoencoder is able to reconstruct the global structure of the input image. Only low-level features at the scale of a few pixels are not precisely captured, causing a slight overall blur in the output.

As described in Section 2.3.1, different compositions across the four data collections (east 2019, west 2019, east 2020 and west 2020) were investigated via clustering in order to better understand the nature of the collected data. In this section, all noise recordings are compared across measurement heights within a certain collection (Figure 7) and across collections with combined heights (Figure 8). For each height in the plots in Figure 7, 250 random linear spectrograms of noise were used to train the autoencoder. The reconstruction quality of the autoencoder is demonstrated in Figure 9. It shows that the trained autoencoder restores all relevant features of an input image up to a resolution of a few pixels. This limitation is commonly observed in encoder–decoder architectures. A quantitative evaluation of the autoencoder is provided in Section 3.1.2. Latent feature representations from another 250 randomly sampled spectrograms for each height are then passed from the trained autoencoder to the UMAP clustering algorithm. The algorithm has two main hyperparameters. One defines the number of neighbors in the temporary state of clustering that are considered to position the next point in the clustering, with emphasis on either global or local features. The other parameter is the minimum distance points are allowed to be apart in the UMAP plot. The best combination of the two hyperparameters is evaluated via grid search for each data setup and shown as a plot in Figure 7 and Figure 8.

For the east 2019, west 2020 and east 2020 collections, a separating clustering is possible for most heights. Some heights partially overlap. Still, different heights correspond to different types of noise (patterns). Only for west 2019, there was no hyperparameter pair showing a clear separation of heights regarding noise. This indicates that noise patterns recorded for west 2019 are more similar to each other across heights than for the other data collections.

When all noise patterns of all collections are put together in one UMAP clustering with combined heights per collection, all collections have a different characteristic noise structure (Figure 8). To be more precise, east 2019 strongly overlaps with west 2019 but also consists of more diverse noise patterns with respect to the overlapping area. Furthermore, east 2019 seems to have some similarities to the manifold of east 2020, indicating potentially similar sources of noise. Interestingly, the noise patterns of west 2020 seem intrinsically different from the rest. A generous visual sampling through the noise data of each collection by eye verified these relations at a higher level. It is important to mention that low-dimensional projections like UMAP cannot reveal all relations at once and cannot focus on small nuances within a spectrogram. Thus, such methods should always be critically evaluated. Taking Table 3 into account, there is no clear explanation for the complexity of noise patterns and their uneven distribution across heights, years and locations. Potential sources of recorded noise seem to be difficult to generalize. In such cases, one must treat the amount of noise data as incomplete with respect to the real-world distribution of noise patterns that are to be expected in this experiment.

#### 3.1.2. Clustering of Genera to Noise Recordings

Interspecific and intraspecific sound variations are caused by multiple disjointed factors [44]. Additionally, echolocation sounds of some genera, like Myotis, Plecotus and the group Nyctaloid, could not be certainly categorized into species via evaluation of audio data by the human expert. Therefore, we recommend investigation of genera and species in separate models. For simplicity, the group Nyctaloid, which also contains several species that have very similar echolocation sounds to Nyctalus species (Table 1), is also treated as a genus class. The autoencoder in this experiment was trained using a range of 100 to 500 examples per genus, along with the noise samples described in Section 3.1.1. To assess the reconstruction quality, a previously unseen test set was utilized, with 20% used as training data. The average PSNR across all classes was found to be 28.448 dB, with a standard deviation of 0.047 dB, indicating good performance. However, the average SSIM value was 0.442, with a standard deviation of 0.006. Visual inspection of the reconstructed images, as shown in Figure 9, revealed a general blur in all examined image pairs. The observed decrease in SSIM can be attributed to this blurring effect, as SSIM is known to be sensitive to structural changes.

**Figure 10:**
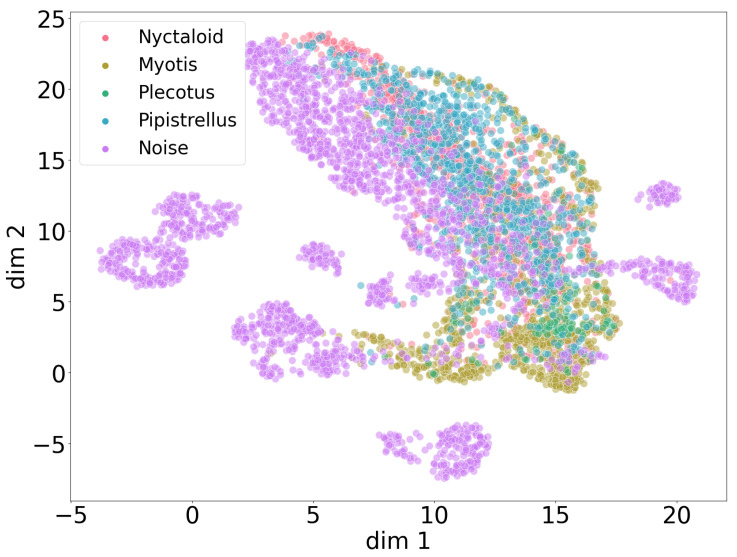
Global UMAP clustering with genus classes and noise recordings over all four data collections. Bat sounds differ from many types of noise patterns but still show similarities to some noise.

The UMAP plot in Figure 10 shows a joined projection of all four genera clustered with all noise patterns from Figure 8. Only the large cluster of noise patterns partially overlaps with the large cluster of the overlapping four genera, revealing that bat sounds usually differ from many types of noise recordings, despite an inherent potential for bat sounds to be confused with other types of noise. We omit an isolated UMAP plot of genera without noise, since it shows no differences in clustering patterns between the genera in the absence of the noise class.

Our unsupervised learning approach performs poorly for the separation of echolocation sounds between species, especially within the same genus. In the case of both versions, with and without a preceding autoencoder, our UMAP algorithm was not able to find a well-separated clustering. A vivid example is given by the spectrogram shown in Figure 1a, which depicts a typical echolocation pulse series of a Pipistrellus bat. Since the autoencoder is fed with the full spectrogram and compresses the image information layer by layer until the bottleneck, to comprehend the general features, it loses essential nuances of the pulses with a comparably short frequency bandwidth and pulse duration. The situation is similar for the UMAP algorithm, which cannot consider such small local information compared to global structures.

### 3.2. Supervised Learning (Cross Validation)

Training and validation sets are from the same collection, i.e., combination of year and location, while the test set is from another collection. Values from the following tables show how certain each class is to be detected on one collection (test) after being optimized on another collection (val). If the performance on the unknown test set is equal to the known validation set, then the general robustness of the model is confirmed for the given circumstances.

#### 3.2.1. Bat/noise Classification (Cross Validation)

In our cross-validation methodology, eight different (directional) combinations of training and test data were investigated, as shown in Table 4. For example, in the first row, the training and validation partition from the east 2019 collection was used to optimize the binary classifier to infer the test partition from the west 2019 collection. The natural imbalance of bat species within a set of bat samples for each collection is maintained for the three partitions (train, val and test) after the split. Based on the findings presented in Section 3.1 about the complexity of the data, especially due to the noise patterns, each collection must be treated as an independent dataset. Therefore, both directions of the investigated collection pairs are taken into account.

The F1 scores for bats seem to be less affected by different years and locations than noise F1 scores. This is likely explained by the fact that bat echolocation sounds are consistent enough across different collections for the model to certainly detect them as bat sounds. As demonstrated in Figure 7 and Figure 8, the variation in noise patterns is likely to be unaffected by the year and location. This is apparently the most intuitive explanation for such strong fluctuations and the reduction in the test F1 scores for noise data shown in Table 4. The F1 score, as shown in Equation (1), takes both classes into account when computing the score for one of the classes. As a consequence, even when almost all bats of the unknown test data are correctly detected, the amount of misclassified noise samples can be detrimental for the F1 score of bats, especially in a balanced dataset of bat and noise samples.

#### 3.2.2. Bat Genus Classification (Cross Validation)

The second version of the CNN is trained for the classification of bat genera. Performance results are shown in Table 5. Fluctuations in performance between the four collections are much less significant than in the bat/noise classification results reported in Section 3.2.1. Therefore, F1 scores for all eight combinations of the four collections as in Table 4 are now averaged for each class (averaged cross validation). As explained in the beginning of Section 3.2, if a class is equally detected for previously seen and unseen data, the general robustness of the model is confirmed for that class. In this case, however, such robustness is only confirmed for the genus Pipistrellus and virtually for Nyctaloid. Nyctaloid shows a slightly reduced F1 score on the test set, averaged across all eight cross validations, with a higher standard deviation as for the validation set. The genus Myotis shows a clear reduction from the train to val set, which may be explained by insufficient training data. It also shows an average decline from the validation to test set but with a similar standard deviation. Both reductions in average performance can also be explained by the general difficulty of detecting Myotis echolocation sounds [48]. In the case of the genus Plecotus, there is a significantly smaller F1 score on the training data compared to the other genera. It additionally shows a drastic decay in validation and test performance, also with high average standard deviations. Taking into account that Plecotus recordings are clearly under-represented in the data for all collections, a lack of features results in the poor Plecotus identification.

#### 3.2.3. Bat Species Classification (Cross Validation)

In this experiment, the CNN is trained for species identification. Since the supervised model described in Section 3.2.2 shows high and, to some extent, robust performance in the case of bat genera, we now focus on the CNN’s ability to discriminate species from the same genus. To this end, we evaluated Pipistrellus as the only genus, providing multiple species in the data. Featured species include *P_pip*, *P_nat* and *P_pyg* as individual species and *P_low* and *P_high* as hybrid classes that contain echolocation sounds of two Pipistrellus species with overlapping frequency ranges. Since the hybrid classes each contain two out of three Pipistrellus species, we train two models (one with and one without the hybrid classes) in order to investigate the impact of such a class format. Unfortunately, the number of examples for the species *P_pyg* is scarce in each collection, leading to an insufficient amount of features to precisely distinguish that species from *P_pip* and *P_nat*. Therefore, we omit the performance values for *P_pyg* in Table 6 and Table 7.

One observation from Table 6 is that the class of *P_pip* is almost perfectly learned by the model, with a validation F1 score of 0.994 and a 0.1% standard deviation. It is also almost fully robust to other collections, with an average F1 score of 0.980 and a 1.6% standard deviation. First, with the classifier performing similarly well on known and unknown data, the model is able to understand the feature space of this class as a whole. Secondly, the feature space of all four collections regarding class *P_pip* is seemingly equivalent, since a classifier is usually not able to extrapolate to unknown features. With 17,000 to 31,000 1 s audio segments for *P_pip* in each collection, the feature space of possible echolocation sounds is sufficiently sampled. For the species *P_nat*, only 600 to 1600 1 s audio segments were recorded per collection. With a significant decline in performance for the validation data, with an F1 score of 0.813 with an 11.1% standard deviation and an average test performance of 0.602 with a 25.7% standard deviation, the characteristic feature space of echolocation sounds for *P_nat* appears to be under-represented by the collected data. With an insufficient database for *P_nat*, it is likely for the model to confuse a certain underlabeled sample of *P_nat* with a similar sound pattern from *P_pip*. This is because all possible echolocation types from *P_pip* seem well-represented by the data, resulting in the identification of uncertain *P_nat* sounds as false-positive *P_pip* sounds.

By adding the hybrid class *P_low* into the training process, the average performance of the classifier for the other classes decreases (Table 7) as a result of class *P_low* comprising sounds from either *P_pip* or *P_nat*, which could not be certainly identified by the human expert via spectrogram evaluation. Since no other classes are involved in that training, the only explanation for a decay in performance for both *P_pip* and *P_nat* is an interaction with *P_low*. Further inspection of the confusion matrix shows that some samples labeled as *P_pip* were identified as *P_low* and vice versa. The same happened between *P_nat* and *P_low*. Hybrid classes, when learned together with examples of their individual classes, are a major source of uncertainty for classification models. Therefore, such classes should be excluded from single-species classification tasks when possible.

### 3.3. Supervised Learning (Complete Data)

This section investigates the ability of the neural network to generalize data that are sampled from the same feature space, i.e., training, validation and test data should be randomly drawn from the same joined dataset. This test should also verify that differences in training, validation and test performance are not caused by a general inability of the model to detect certain types of features that may be present in only some classes. Therefore, all four collections are joined into one complete dataset, then split into three partitions as training, validation and test data with a ratio of 60:20:20. During this split, each class is randomly and individually sampled in order to maintain the natural imbalance of the whole dataset from before the split. This also guarantees that rare species are equitably represented in each of the three partitions. A joined training for the task of bat/noise classification is currently omitted, since no relation can be found between the background noise structure and location, year and measurement height of a recording (Section 2.2.3). Thus, a joined model is still not guaranteed to be provided with the complete feature space of noise patterns.

#### 3.3.1. Bat Genus Classification (Complete Data)

The average model performance of the classifier is depicted in Table 8. When the model is trained on the full data, combining all four collections into one set, it performs equally well on known validation data and unknown test data. This is proven by equal F1 scores for validation and test sets. Compared to Table 5, which shows average performance from cross validation, the validation F1 score of Myotis is significantly higher when the data are joined across all collections. This means that the variety in features for Myotis sounds is improved by joining the collections from different locations and times. Only the genus Plecotus is still not provided with sufficient data.

#### 3.3.2. Bat Species Classification (Complete Data)

Improvements similar to the bat genus classification presented in Section 3.3.1 are also achieved for bat species classification. By comparing Table 9 with Table 6, one can see an unchanged level of performance for validation and test data in Table 9. This proves that the model is able to fully comprehend the feature space of the complete dataset, making it robust to unknown test data drawn from the same feature space.

When the classifier is trained with the hybrid class *P_low*, a reduced average performance is still measurable in the case of the complete data (Table 10). However, it performs better than in the cross-validated approach listed in Table 7.

**Figure 11:**
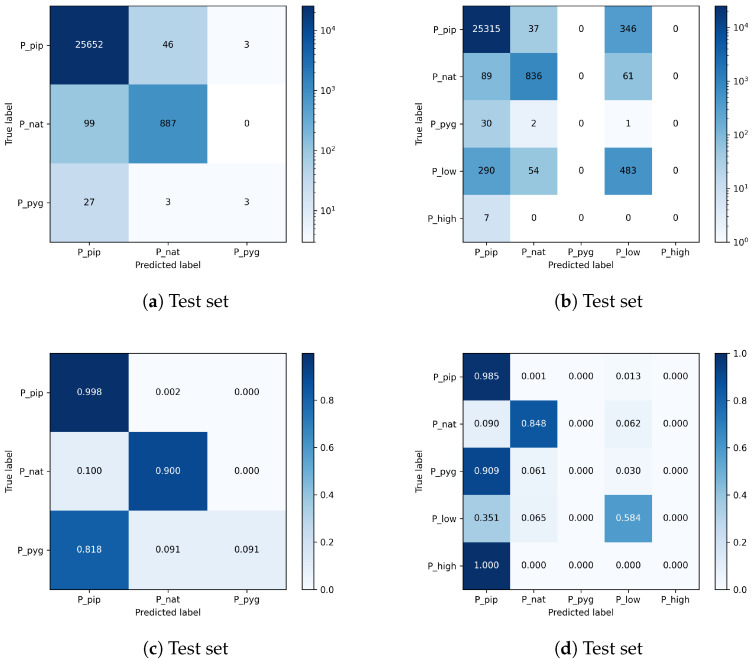
Confusion matrices for species classification with three classes or five classes. Results are averaged across five runs. Including the hybrid class *P_low* in the optimization process induces uncertainty and causes the network to confuse samples with the individual species, especially *P_pip* and *P_nat* and vice versa. The top matrices show the absolute numbers and a logarithmic color normalization across all entries. The bottom two matrices provide the relative numbers color-coded and normalized for each class.

A better understanding of the impact of a hybrid class, which induces additional uncertainty in model decisions, is provided by the confusion matrices in Figure 11. Figure 11a contains the average confusion matrix of the model trained without the hybrid class *P_low*, and Figure 11b is of the model trained with hybrid class *P_low*. The results show that a significant amount of bat sounds labeled as *P_pip* or *P_nat* are classified as *P_low* and vice versa. The bat species class *P_pyg* and the other hybrid class *P_high* are trained by the classifier and are also covered in the confusion matrices but have strongly insufficient data. Thus, these two classes are omitted in Table 9 and Table 10 for simplicity.

## 4. Discussion

The idea of using an autoencoder to improve the convergence speed of UMAP comes from its ability to compress a single image by adapting to the whole dataset (Figure 5). Other standard (lossy) image compression algorithms are probably more efficient but less dynamic and usually do not consider the whole dataset in order to compress individual images.

A quantitative comparison of our model was only possible with of Schwab et al. [35] because comparable methods like those of Chen et al. [33], Kobayashi et al. [34] and Tabak et al. [36] have recorded different bat species due to their measurement locations. The distribution of bat species investigated in Schwab et al. [35] has a strong intersection with our dataset. Therefore, we computed the accuracies for our two species (*P_pip* and *P_nat*), the genus (*Myotis*) and the group (*Nyctaloid*). To facilitate a comparison between our results for *Myotis* and *Nyctaloid* with the findings of Schwab et al. [35], we calculated the average accuracies of their best model specifically for the species present in our *Myotis* and *Nyctaloid* class, as listed in Table 1.

Table 11 demonstrates a slightly higher performance compared to that obtained by Schwab et al. [35], but this comparison must be considered under the following circumstances. First, both works investigated a different dataset, and the quality and difficulty of the data have a strong impact on the final model performance. With our model learning the full frequency range of the raw audio data, we argue that our model performs well under real-world conditions. Second, Schwab et al. [35] trained a single model with 18 species. Although our work trained a different model for species and genera, we argue that our model is, by design, still able to handle a similar number of classes at once.

Our classifier was designed in favor of future edge-AI applications. The simplicity of the main classification network (Figure 6) still provides very high predictive capabilities as long as the natural intrinsic difference in sounds between the defined classes is provided under the additional premise of sufficient data volume. We show that the low complexity of the classification network is sufficient to allow for highly discriminative performance for all levels of classification from bat/noise classification to genus and even species classification (Section 3.2 and Section 3.3). Our work, unlike other studies mentioned in Section 1, demonstrates that overly complex neural networks are not always necessary for bat echolocation sound analysis. For example, Schwab et al. [35] used a modified ResNet-50, a network with 49 convolutional layers, as their best model. A network with that many layers is overly large, takes too long to train and fine tune and usually does not fit on low-power devices like microcontrollers. The variation in geometric patterns of bioacoustic data is much less compared to generic datasets such as ImageNet or MS-Coco. A large number of channels in convolutional layers becomes unnecessary and memory-expensive, since the model focuses on the relevant features of the input data. Many channels are then likely to be filled with redundant or even insignificant features. When implemented in a smaller framework, like TinyML or TensorFlow Lite, our model is fit for deployment on low-power devices such as microcontrollers and AI chips similar to that reported by Zualkernan et al. [49].

Paumen et al. [38] indicated that a classifier trained on a single recording height is able to safely detect bat sounds recorded at the other heights from the same year and location. On the other hand, a significant amount of noise samples from other heights is misclassified as bat sounds [38]. The results reported in Section 3.2.1 reveal similar behavior in our tests on noise recordings from data collections unknown to the optimized classifier for bat/noise classification. A more distinctive comparison of evaluation metrics with respect to single bat classes in the bat/noise classification in Section 3.2.1 may reveal new reasons for the observed behavior, but this is beyond the scope of this work and requires more examples for rare species to be authentic.

Works evaluated in Section 1 usually focused on optimizing a single model that learns a joined dataset from all sources or sites. Their goal is to maximize their discriminative model performance, as in Section 3.3. Instead, we invested additional effort to further understand the impacts of conditional factors on the data and therefore the model robustness and generalization ability, as shown in Section 3.2. The methods reported by Chen et al. [33], Kobayashi et al. [34], Schwab et al. [35] and Aodha et al. [31] only use small windows of a maximum pf 27 ms as input data for the neural network. Such short audio segments contain one pulse, at most. Instead, our model implicitly comprehends the common interpulse interval of a species in a 1 s MFCC without the need for manual extraction of this feature. The interpulse interval is a primary indicator in manual echolocation sound identification. Therefore, our model can provide a practical advantage over previously mentioned methods.

There is always the question of how to compare human cognitive performance with the performance of computational statistical models like neural networks. First, when a model is trained by human-labeled data, the labels are treated as correct by the model, i.e., the supervised model tries to imitate the labeling behavior of the human. Second, a neural network has a more rigid understanding of a class compared to a human brain. Human experts, as in our case, are trained to comprehend such audio data with multiple sources of knowledge or experience, which is usually a composition of various sensations.

In order to improve the robustness of the model and its ability to generalize well on the data, datasets may be improved in several ways. One approach is to extend the audio database of bat sound recordings. This can be managed by unifying multiple sources from different locations and audio recording devices shared between research organizations, as reported by Görföl et al. [50]. Another way of improving the audio database is to consider habitat influences and corresponding bat sounds as reported by Findlay et al. [51]. Inspired by the way humans perceive the world via multiple senses, it may be advisable to combine acoustic monitoring with video tracking of bat activity as reported by Thomas et al. [52]. Examination of carcasses in areas where populations are tracked can help to offer probabilities of occurrence of different species for detection models as reported by Chipps et al. [53].

## 5. Conclusions

This work introduces a novel approach for investigating and identifying real-world bat echolocation sound recordings. This study demonstrates that more authentic interpretations of auditory data can be made with the help of unsupervised learning methods like autoencoders and UMAP clustering. The following specific insights contribute to the research areas of bat echolocation sound analysis, acoustic monitoring and bat conservation.

Noise recordings that exceed the threshold parameters of devices like the BATmode S+ system can be of various forms and origin. They are difficult for the model to generalize on as long as their origin and full extent remain unknown, as revealed by UMAP clustering and a preceding convolutional autoencoder used for image compression. Neither the autoencoder or the UMAP algorithm are designed to comprehend low-level features from data samples, which explains their ineffectiveness on bat species of the same genus.

In the case of bat/noise classification, if a pretrained classifier is applied to unknown data from a different feature distribution, the detection of bats is much more reliable than the detection of noise. This is explained by the complexity of noise patterns and their unknown origins.

Bat genus classification and bat species classification experiments show that the performance of the classifiers in detecting genera and species depends on the location of measurements. Its performance is even influenced by the time of recordings, which is likely explained by environmental changes over time. Our results also indicate that after a certain number of examples of echolocation sound segments from all locations and years, any of the classes can be fully comprehended by the proposed model.

Moreover, the obtained results constitute practical proof that short, standard CNNs are fully sufficient for classifying bat echolocation sounds at the genus and species levels, making them attractive for edge-AI applications on low-power devices.

Most bat sound identification software is only commercially available, which may unnecessarily decelerate the progress in the field of bioacoustics. By providing an open-source version of our code, we intend to simplify the deployment of bat identification projects by shortening the initial time and financial resources required to perform first classifications.

## Figures and Tables

**Table 1:** Overview of bat classes with descriptions of each class. The expected species for each genus and group are supported by geodata from the Landesanstalt für Umwelt Baden-Württemberg (LUBW) [45].

Class	Definition	Description
P_pip	Species *Pipistrellus pipistrellus*	Frequency of maximum energy (peak): 42 to 50 kHz
P_nat	Species *Pipistrellus nathusii*	Frequency of maximum energy (peak): 35 to 40 kHz
P_pyg	Species *Pipistrellus pygmaeus*	Frequency of maximum energy (peak): 52 to 60 kHz
N_noc	Species *Nyctalus noctula*	Frequency of maximum energy (peak) <20 kHz
B_bar	Species *Barbastella barbastellus*	
Myotis	Genus *Myotis*	Species are difficult to distinguish; strong overlap of call parameters; possible species: *Myotis bechsteinii*, *Myotis daubentonii*, *Myotis myotis*, *Myotis mystacinus*, *Myotis nattereri* and *Myotis brandtii*
Plecotus	genus *Plecotus*	Species are difficult to distinguish; strong overlap of call parameters; possible species: *Plecotus auritus* and *Plecotus austriacus*
Nyctaloid	group *Nyctaloid*	Species are difficult to distinguish; strong overlap of call parameters; possible species: *Nyctalus noctula*, *Nyctalus leisleri*, *Eptesicus serotinus*, *Eptesicus nilssonii* and *Vespertilio murinus*
P_low	Species *Pipistrellus pipistrellus* Species *Pipistrellus nathusii*	Includes echolocation sequences of both species for an intersecting frequency range from 40 to 42 kHz
P_high	Species *Pipistrellus pipistrellus* Species *Pipistrellus pygmaeus*	Includes echolocation sequences of both species for an intersecting frequency range from 50 to 52 kHz

**Table 2:** Bat sound segments from four data collections. All four measurement heights are joined and recycled in 1 s intervals.

Class	East 2019	West 2019	East 2020	West 2020
P_pip	17,761	22,801	20,093	31,336
P_nat	723	855	611	1607
P_pyg	35	28	29	15
N_noc	7697	10,765	3151	9767
B_bar	13	21	93	185
Myotis	2987	4439	995	4884
Plecotus	24	42	93	406
Nyctaloid	6549	12,625	4601	28,087
P_low	629	799	364	1186
P_high	0	3	11	1

**Table 3:** Overview of noise recordings from four data collections.

Height of Measurement	East 2019	West 2019	East 2020	West 2020
10 m	34,481	33,466	118,692	443,609
35 m	630	250	6412	7903
65 m	256	191	2545	7074
95 m	10,507	199	3095	2206

**Table 4:** Bat/noise classification. Cross validation over four data collections. All four heights of measurement are joined within a data collection of year and location. The F1 score of each class represents the average performance on the test data from five runs.

Direction (Train → Test)	F1 Score (Bat)	F1 Score (Noise)
East 2019 → West 2019	0.994 ± 0.002	0.978 ± 0.008
West 2019 → East 2019	0.842 ± 0.007	0.700 ± 0.020
East 2020 → West 2020	0.927 ± 0.041	0.769 ± 0.178
West 2020 → East 2020	0.805 ± 0.057	0.614 ± 0.149
East 2019 → East 2020	0.884 ± 0.017	0.789 ± 0.041
East 2020 → East 2019	0.856 ± 0.058	0.768 ± 0.131
West 2019 → West 2020	0.876 ± 0.014	0.446 ± 0.100
West 2020 → West 2019	0.937 ± 0.033	0.616 ± 0.261

**Table 5:** Bat genus classification. Averaged cross validation over four data collections with five runs each. All four heights of measurement are joined within a data collection of year and location.

Class	F1 Score (Train)	F1 Score (Val)	F1 Score (Test)
Nyctaloid	0.996 ± 0.004	0.957 ± 0.027	0.893 ± 0.076
Myotis	0.944 ± 0.096	0.862 ± 0.154	0.696 ± 0.168
Plecotus	0.522 ± 0.356	0.263 ± 0.331	0.139 ± 0.202
Pipistrellus	0.999 ± 0.001	0.991 ± 0.004	0.970 ± 0.013

**Table 6:** Bat species classification without hybrid class *P_low*. An averaged cross validation is performed over four data collections with five runs each. All four heights of measurements are joined within a data collection of year and location. *P_nat* shows insufficient data for cross validation.

Class	F1 Score (Train)	F1 Score (Val)	F1 Score (Test)
P_pip	0.999 ± 0.001	0.994 ± 0.001	0.980 ± 0.016
P_nat	0.983 ± 0.018	0.813 ± 0.111	0.602 ± 0.257

**Table 7:** Bat species classification with hybrid class *P_low*. An averaged cross validation is performed over four data collections with five runs each. All four heights of measurement are joined within a data collection of year and location. The results demonstrate a decline in performance for *P_pip* and *P_nat*, since *P_low* contains pulse shapes of both species at a mutual frequency level. Uncertainty is introduced by class *P_low*.

Class	F1 Score (Train)	F1 Score (Val)	F1 Score (Test)
P_pip	0.997 ± 0.002	0.981 ± 0.004	0.964 ± 0.013
P_nat	0.881 ± 0.101	0.681 ± 0.178	0.479 ± 0.200
P_low	0.919 ± 0.043	0.402 ± 0.089	0.226 ± 0.074

**Table 8:** Bat genus classification. A single model is trained on all four data collections. All four heights of measurement are joined within a data collection of year and location. F1 scores are averaged from five runs. The results show strong predictive capabilities for a genus when sufficient data are provided.

Class	F1 Score (Train)	F1 Score (Val)	F1 Score (Test)
Nyctaloid	0.996 ± 0.002	0.979 ± 0.001	0.978 ± 0.002
Myotis	0.996 ± 0.002	0.947 ± 0.003	0.946 ± 0.003
Plecotus	0.793 ± 0.441	0.566 ± 0.317	0.552 ± 0.310
Psuper	0.999 ± 0.000	0.994 ± 0.001	0.994 ± 0.001

**Table 9:** Bat species classification without overlap class *P_low*. A single model is trained on all four data collections. All four heights of measurement are joined within a data collection of year and location. F1 scores are averaged from five runs. The results show strong predictive capabilities for a species when sufficient data are provided.

Class	F1 Score (Train)	F1 Score (Val)	F1 Score (Test)
P_pip	1.000 ± 0.000	0.996 ± 0.000	0.997 ± 0.000
P_nat	0.993 ± 0.006	0.919 ± 0.005	0.923 ± 0.009

**Table 10:** Bat species classification with overlap class *P_low*. A single model is trained on all four data collections. All four heights of measurement are joined within a data collection of year and location. F1 scores are averaged from five runs. The results demonstrate a decline in performance for *P_pip* and *P_nat*, since *P_low* contains pulse shapes of both species at a mutual frequency level. Uncertainty is introduced by class *P_low*.

Class	F1 Score (Train)	F1 Score (Val)	F1 Score (Test)
P_pip	0.995 ± 0.001	0.984 ± 0.001	0.984 ± 0.001
P_nat	0.975 ± 0.004	0.871 ± 0.017	0.873 ± 0.012
P_low	0.875 ± 0.022	0.558 ± 0.013	0.562 ± 0.011

**Table 11:** Performance comparison with Schwab et al. [35]. Other classes are omitted due to a lack of samples in our training data.

Class	Accuracy (Schwab et al.)	Accuracy (Ours)
P_pip	0.985	0.997
P_nat	0.889	0.994
Myotis	0.983	0.986
Nyctaloid	0.950	0.986

## Data Availability

The dataset analyzed in this study is restricted by the German Federal Agency for Nature Conservation (German: Bundesamt für Naturschutz, BfN). The complete programming source code is publicly available from Mendeley Data under DOI:10.17632/9x2g6dsbtv.1 https://data.mendeley.com/datasets/9x2g6dsbtv (accessed on 31 July 2023). Versions of relevant code packages and frameworks are listed in the repository. The code is applicable to similar acoustic bat datasets.

## Abbreviations

The following abbreviations are used in this manuscript:

CNN	Convolutional neural network
UMAP	Uniform manifold approximation and projection
STFT	Short-time Fourier transform
FFT	Fast Fourier transform
MFCC	Mel frequency cepstral coefficient
